# Access to and use of health and social services among people who inject drugs in two urban areas of Mozambique, 2014: qualitative results from a formative assessment

**DOI:** 10.1186/s12889-020-09068-8

**Published:** 2020-06-22

**Authors:** Liliana Dengo-Baloi, Makini Boothe, Cynthia Semá Baltazar, Isabel Sathane, Denise Chitsondzo Langa, Manuel Condula, Helena Ricardo, Celso Inguane, Eugénia Teodoro, Lídia Gouveia, Henry F. Raymond, Roberta Horth

**Affiliations:** 1 Instituto Nacional de Saúde, Maputo, Mozambique; 2 University of California, San Francisco, USA; 3 Faculty of Medicine and Health Sciences, Ghent University, Ghent, Belgium; 4 National HIV/STI Control Program, Public Health Directorate, Ministry of Health, Maputo, Mozambique; 5 Rede Nacional Contra Drogas (UNIDOS), Maputo, Mozambique; 6 Department of Anthropology, University of Washington, Seattle, USA; 7 Mental Health Department, National Public Health Directorate, Ministry of Health, Maputo, Mozambique; 8 School of Public Health, Rutgers University, Piscataway, NJ USA

**Keywords:** PWID, Health access, Mozambique, HIV key population

## Abstract

**Background:**

Prior to 2014, data about health seeking behaviors or service uptake for People who inject drugs (PWID) in Mozambique did not exist. We present the results from the formative assessment component of the Biological and Behavioral Survey (BBS).

**Methods:**

Standardized interview guides were used during key informant interviews (KII) and focus group discussions (FGD) in Maputo and Nampula/Nacala to discuss issues related to risk behaviors and access to and utilization of health and social services by PWID. The target sample size was not defined a priori, but instead KII and FGD were conducted until responses reached saturation. Data analysis was based on the principles of grounded theory related to qualitative research.

**Results:**

Eighty-eight respondents, ages 15 to 60, participated in KIIs and FGDs. Participants were majority male from diverse income and education levels and included current and former PWID, non-injection drug users, health and social service providers, peer educators, and community health workers. Respondents reported that PWID engage in high-risk behaviors such as needle and syringe sharing, exchange of sex for drugs or money, and low condom use. According to participants, PWID would rather rent, share or borrow injection equipment at shooting galleries than purchase them due to stigma, fear of criminalization, transportation and purchase costs, restricted pharmacy hours, personal preference for needle sharing, and immediacy of drug need. Barriers to access and utilization of health and social services include distance, the limited availability of programs for PWID, lack of knowledge of the few programs that exist, concerns about the quality of care provided by health providers, lack of readiness as a result of addiction and perceived stigma related to the use of mental health services offering treatment to PWID.

**Conclusions:**

Mozambique urgently needs to establish specialized harm reduction programs for PWID and improve awareness of available resources. Services should be located in hot spot areas to address issues related to distance, transportation and the planning required for safe injection. Specific attention should go to the creation of PWID-focused health and social services outside of state-sponsored psychiatric treatment centers.

## Introduction

The use of injectable drugs is a known risk factor for the spread of Human Immunodeficiency Virus (HIV) and other blood borne diseases [1]. In addition to socioeconomic and legal challenges, people who inject drugs (PWID) experience a range of health problems such as increased mortality compared with the general population [1]. The United Nations Office on Drugs and Crime (UNODC) estimated in 2016 that there were 10.6 million PWID worldwide, where one out of eight PWID are living with HIV [1]. In sub-Saharan Africa, UNODC estimated that the average prevalence of HIV infection among PWID was around 11% [1]. Given that injection drug use is increasingly common among young adults in sub-Saharan Africa and that drug use is also associated with increased sexual risk behaviors, PWID are known to play an important role in generalized HIV epidemics in the region [2]. Harm reduction programs, based on effective substance use education and drug treatment services, are very limited, while imprisonment, due to the criminalization of drug use, is common [2].

Studies have identified various structural barriers to access to health services in sub-Saharan Africa such as criminalization of drug use, punitive laws, uncoordinated services, lack of transportation, high cost of services and fear of stigmatization [2]. Other barriers include systemic inequalities, such as discrimination, as well as intrapersonal factors, such as the preference of PWID to use addictive substances rather than seeking medical help [3]. Other studies have pointed to barriers such as the restriction of treatment to in-patient services, which limits acceptability of such services, as well as the difficulty in meeting entry requirements [4].

Prior to 2013, there was no data about health seeking behaviors or service uptake specifically for PWID in Mozambique. Government interventions for this population mainly focused on educational campaigns to prevent drug use, and the provision of mental health services and psychiatric treatment for PWID at public health facilities [2]. Interventions also included HIV counseling, testing and linkage to care at health facilities [5]. However, needle and syringe exchange programs and opioid substitution therapy - key components of national harm reduction strategies - were not available in Mozambique [1]. There is a critical need to describe the health seeking behaviors and barriers to care among this population in order to inform the design of effective interventions and evidence-based policies.

The first Biological and Behavioral Survey (BBS) among PWID in Mozambique was conducted from 2013 to 2014 to address this gap in knowledge. The purpose of the survey was to estimate the prevalence of HIV, hepatitis B and hepatitis C among PWID in two urban areas in Mozambique (Maputo City and Nampula City / Nacala City) and to identify associated risk behaviors [6]. In addition, the survey assessed access to and use of health and social services for PWID in those urban areas.

Before survey implementation, a formative assessment was conducted to inform the design and implementation of the BBS survey using qualitative research methods and tools common to ethnographic research. A Formative Assessment is an important element of any BBS survey because it provides the design and implementation of the survey and also establishes a relationship between the research team and key stakeholders. According to the WHO, the Formative Assessment is exploratory and multimethod with the following objectives: (a) understand the target population and context; (b) identify existing services and gaps; (c) inform survey methods (e.g. sampling strategy, procedures, questionnaire and biological specimen collection); and (d) engage stakeholders [7, 8]. The Formative Assessment included two phases: [1] ethnographic observation and mapping and [2] stakeholder engagement through semi-structured interviews with key informant and focus group discussions. We present the results of the key informant interviews and focus group discussions to assess access to and use of health and social services among PWID [8].

The purpose of the formative assessment was to examine the logistics of carrying out this Integrated Biological and Behavioral Survey (IBBS) and assess the characteristics of PWID, locations these populations often visit, availability of social welfare and health services, and other information particular to this key population. We present the results of the formative assessment of the IBBS among PWID related to access to and use of health and social services.

## Methodology

The formative assessment was conducted in Maputo City and Nampula City/Nacala City between June and July 2013 and included key informant interviews (KII) and Focus group discussions (FGD) with former and current PWID and people knowledgeable about the target population such as members of organizations providing services targeted to PWID (Nampula City and Nacala City was considered one study site for having the same drug users network). KII and FGD were conducted until responses reached data saturation – a point at which further data collection is unlikely to produce any new information [9]. All KII and FGD followed a semi-structured interview format and were conducted in Portuguese.

### Key informant interviews

The key informants served as “community experts” who could provide insight into the risk behaviors of PWID and their access to health and social services. These key informants were a diverse group that included community leaders, current and former PWID, lay counselors, community activists, peer educators, current drug dealers, researchers working with PWID, health professionals and other service providers [10]. The information gathered through KII was used to formulate FGD questions to explore issues in greater depth.

### Focus group discussions

The FGD were conducted by two trained research assistants who served as moderator and annotator [11]. The FGD were designed to provide information on topics of interest such as risk behaviors in the community of interest and social networks of PWID. Recruitment of FGD participants was done through purposive sampling to provide a comprehensive description of PWID in each study location [12].

### Data collection tools

Standardized interview guides were used during KII and FGD to facilitate discussion of issues related to socio-demographic characteristics of PWID, risk behaviors, access to and use of health services, barriers to the provision of health care, and social assistance offered to PWID. The interview guide has been previously published [13] The acceptability of the BBS research procedures such as the proposed sampling method, biological testing and treatment for sexually transmitted diseases (STIs) and blood borne diseases were also discussed, however these results are beyond the scope of the current manuscript.

### Data analysis

All KIIs and FGDs were audio recorded and transcribed. Field notes were developed immediately after each KII and FGD and later transcribed. Data analysis was based on the principle of grounded theory and aligned with data analysis procedures by the WHO for formative assessments [7], whereby codes were grouped into categories and then categories grouped together to form themes [11]. Transcripts were coded and analyzed using the qualitative research software application NVivo Version 10 (QSR International Pty Ltd.). Two study investigators independently coded the transcripts and compared codes. Any ambiguities or discrepancies in coding were discussed and resolved. Categories and themes were also determined jointly. For the purpose of this manuscript, quotes were translated into English by two co-authors fluent in both English and Portuguese and then verified by other bilingual co-authors.

## Results

We conducted 22 key informant interviews and 4 FGD (with 6–12 participants for each group), including a total of 40 FGD participants (Table 1). The majority of participants were male (51 male and 9 women) and represented different socioeconomic and educational levels. Those who participated in FGD or KII came from a variety of backgrounds including current and former PWID, health and social services, and peer educators/community health workers. KII participants were representative of several organizations such as drug abuse prevention services, faith-based organizations, social reintegration services, psychological and psychotherapeutic assistance, treatment organizations, and government institutions.

**Table 1:** Description of Key informants and associations that participated in the qualitative assessment on barriers to access to health and social services for people who Inject Drugs in the cities of Maputo and Nampula/Nacala, Mozambique, 2013

Maputo	Nampula/Nacala
Youth Association	Health Facility
Hospital-based out-patient support group	Out-patient psychiatric services
Community Association	Out-patient psychiatric services
Association for social rehabilitation	Peer Educator
NGO for Health Communications	Association for social rehabilitation
Association for Family Aid	Current drug dealer
Government agency for drug control and prevention	Out-patient psychiatric services
Psychiatric Hospital	Hospital
Rehabilitation Center	Current PWID
Out-patient psychiatric services	Association for social rehabilitation
Former PWID	
Current PWID	

### Sociodemographic characteristics of PWID

Participants reported that PWID were a diverse group between the ages of 15 and 60 years, the large majority of whom were male, from all income levels, and spoke various national, regional and global languages. A participant described the diversity of socioeconomic status among PWID, as follows:

I can see people who consume [drugs] that have limited financial resources, so poor. Sometimes I do not know how they can afford the drugs they consume. And also, there are people of high social level who are consuming cocaine, even injecting heroin [...]

Participants reported that although some PWID have regular jobs and can in some way afford drugs, others often have unstable economic conditions and are eventually involved in unlawful activities to support their drug use:

There are those who live from garbage bins, there are those who are street vendors, there are those who sell second hand clothes, there are street burglars, there are others who use knives to steal money, there are others who steal clothes in buildings, there are others who steal bottles, there are others who have money because of work, there are scammers, there are taxi drivers, there are the children whose papa and mama give them money [...]

### Sexual risk behaviors

There was a high level of sexual risk behaviors reported by participants, particularly low condom use among PWID often due to a preference for “*carne com carne*” (flesh on flesh, a term commonly used to describe sex without a condom). It was reported that drug use impaired condom use: “You need to remember that after consuming drugs, you see Jesus, so just imagine, after seeing Jesus, you don’t have time to use a condom.”

Participants also mentioned sex work as a common practice among PWID. Women who inject drugs were reported to trade sex for drugs or for money to buy drugs. As one participant mentioned, “girls end up in prostitution to support their habit because they have no easy access to money.” However, sex work was not perceived to be exclusively associated with women who inject drugs.

Often, gays in their fancy cars go to the places frequented by the PWID and call one and ask if they want cigarettes. They then invite them to stroll and go to a discrete location. Here, they propose to trade sex for money. When the act takes place, these men do not pay little, they pay well [...]

### Access to injection materials and needle sharing

Participants mentioned that although PWID had knowledge of where to access clean needles, needles were often bought through illicit means because of stigma and fear of criminalization:

And even dealers themselves purchase a certain amount of syringes and you purchase the syringe there, done. This [buying syringes from dealers] is more for people who do not want anyone to find out that, ‘wow … he’s doing these things’ (i.e.: injecting drugs).

[...] The syringes are rented for about 50 meticais (<$2 USD) in crack houses (houses where people sell or do drugs), because it is safer, instead of buying the [syringe for] 10 meticais (<$0.40 USD) in pharmacies and then hanging around and risk of police finding and arresting the person on charges of being under the influence of drugs.

In both study sites, it was also reported that health professionals often trade new and/or used syringes from health facilities: “There are syringes sold by employees of health facilities. Some employees collect used syringes that should be disposed off in the trash, and sell them.”

Needle sharing and exchange was also commonly reported:

In the crack houses, [renting of syringes] happens “without a lease,” meaning that no one questions whether the syringe and needle is sterile or not.

“One thing I can be sure of, when there are no syringes, a single syringe serves for many users, they do not take care of themselves very well, so the risk is still there.”

Despite knowledge of where to obtain clean needles, sharing was reported to be a result of barriers to transportation, as one participant explained:

[If] I’m in need at this time … I’m having withdrawal symptoms and I have to run up there to the pharmacy, take public transport, go to the pharmacy [that’s] closed from 12 pm to 2 pm, to be able to get another syringe and then go to inject myself … it’s easier to take another [needle, that someone else has used] and use it, because it has an immediate effect [of getting me high].

### Barriers to access and use of health and social services

The need for health and social services for PWID was mentioned during both KII and FGD especially given that this population often suffers from problems resulting not only from drug use but also from associated risk behaviors and poor living conditions. As mentioned in a FGD: “Many of us, who are drug users, right, we have health problems, yeah, and HIV, TB … ”.

Participants also perceived there to be a shortage of PWID-targeted programs. Respondents often indicated that the lack of treatment options forced them to end their drug addiction abruptly (“cold turkey”,) without opioid replacement therapy to reduce withdrawal symptoms.

Some respondents felt that HIV counseling and testing services have to be more accessible in terms of location and working hours. For PWID who were aware of health services, the poor quality of these services was a barrier to health-seeking behavior:

[T] here are centers that do not treat us well. For example, there is bad food and when one goes to be hospitalized, but then sees the conditions, they can’t endure the time required to get better.

### Structural barriers

The distance between where PWID live and socialize and health care and social services was another barrier to the access and use of services. One respondent noted, “The problem is the characteristic of this group. People are closed off (isolated in certain areas) so it automatically gets complicated for them to go to an institution [providing services].”

Participants also mentioned other structural barriers, some of which were specific to the Mozambican context, where services cannot be given anonymously and an identity document must be presented to receive services: “For example, I don’t go to the hospital because I have no (identity) document.”

This structural barrier was linked to the fear of potential criminalization:

The first thought is that there is lack of trust for these people [health workers] … they want to collect information about us that can be referred to the police about where we are to later cause problems.

Finally, the participants mentioned that state-sponsored treatment services located at psychiatric hospitals was a barrier to use of such services because of the stigma associated with mental illnesses:

I said yes, I am a drug user … [and] they sent me to the psychiatric hospital and I thought, ‘the mental hospital is a hospital for crazy people and I’m not crazy.’

[T] he only hospital that provides such services is the psychiatric hospital. The psychiatric hospital deters young PWID because they have the fear of being labeled as mentally ill, crazy and somehow end up crazy.

PWID also mentioned their experience with stigma from healthcare providers or centers as a barrier to care:

So, these are the conditions we need in a health center. We got there, we are well received as anyone else, but because there are those who go there and ... you know, “this is a junkie or because he is poorly dressed” or because he appears to be half dizzy and treatment is done... but not equally [to other patients]. So these are things [equal treatment] we also need as drug addicts.

Finally, the lack of one-stop services was mentioned as a barrier where PWID had to ask “several times for support” at a centralized location. One key informant described the long referral process and the resulting gap in linkage to services:

The referral process for an injection drug user is very complicated because he can receive the referral document and throws it away, never going to the hospital … To facilitate my work, I prefer to go with him to the hospital because if you send them he won’t go and will end up prioritizing something else.

### Facilitators of access and use of health and social services

Despite most participants being dissatisfied with the quality of services received, some described a positive experience with the health system. One of them reported, “in my case for example, I had treatment for about six months or something when I contracted tuberculosis, years ago, and we were well attended”.

One participant noted that a staff member from a treatment organization explained the purpose of treatment in order to address the stigma associated with the use of psychiatric services:

[I] t required a deep conversation for him (the staff member from a treatment organization) to explain that no, the hospital is not for crazy people but that (drug use) is a type of mental illness. So eventually, I accepted and went there.

Participants mentioned the existence of different institutions and organizations where they could obtain physical and mental health support, which included disease and drug abuse prevention, peer educators, counseling and public informational meetings; voluntary HIV counseling and testing at health facilities; psychosocial support including psychological and psychotherapeutic assistance for detox; social reintegration through the development of professional skills and competencies, and social re-integration programs (Table 2).

**Table 2:** Organizations providing social and health services to the PWID by province, identified by participants in the qualitative assessment on barriers to access to health and social services for people who inject drugs in the cities of Maputo and Nampula city/Nacala, Mozambique, 2013

Province	Organization	Services
Maputo	NGO	Disease and drug abuse prevention^**a**^
	NGO	Disease and drug abuse prevention ^**a**^
	NGO	Disease and drug abuse prevention ^**a**^
	NGO	Disease and drug abuse prevention ^**a**^
	NGO	Disease and drug abuse prevention ^**a**^
	NGO	Disease and drug abuse prevention ^**a**^
	NGO Treatment Centre	Spiritual Rehabilitation ^**b**^
		Social Reinsertion ^**c**^
	NGO	Disease and drug abuse prevention ^**a**^
	Public Treatment Centre	Psychological and Psychotherapeutic assistance ^**d**^
		Referrals for health services
	Public Treatment Center	Psychological and Psychotherapeutic assistance ^**d**^
		Referrals for health services
Gaza	NGO Treatment Centre	Spiritual Rehabilitation ^**b**^
		Social Reinsertion ^**c**^
Inhambane	NGO Treatment Centre	Spiritual Rehabilitation ^**b**^
		Social Reintegration ^**c**^
	NGO	Disease and drug abuse prevention ^**a**^
Sofala	NGO Treatment Centre	Spiritual Rehabilitation ^**b**^
		Social Reintegration ^**c**^
Nampula	NGO	Disease and drug abuse prevention ^**a**^
	Public Treatment Unit	Psychological and Psychotherapeutic assistance ^**d**^
		Referrals for health services
	Public Treatment Unit	Services on HIV testing and counselling
Cabo Delgado	NGO	Disease and drug abuse prevention ^**a**^

^**a**^Illicit drug use and disease prevention through primary prevention services

^**b**^Spiritual rehabilitation is based on religion beliefs and faith

^**c**^Social reinsertion consists of providing training for ex-PWID to find an occupation

^**d**^Psychotherapy and psychological assistance consists of out-patient appointments with a trained mental health professional and pharmaceutical treatment to fight addiction

A key informant, who worked as a service provider, described a comprehensive list of services offered by a local institution:

This institution [is a] rehab center based on a spiritual care program, and also some other extra activities such as occupational therapy and vocational training which are included in various activities such as car repair/mechanic activities, [for] example. You know, therapy for a process of transformation and also capacity building so people can be self-sustainable.

## Discussion

The socio-demographic characteristics of PWID as described by our participants was similar to previous studies, including diverse age range and economic backgrounds and mostly male [2]. The exchange of sex for drugs or money to buy drugs was also described as a common practice, especially among female injection drug users. This same dynamic was observed in Ghana [14] where female PWID reported exchanging sex for money and/or drugs and male PWID reported paying for sex from female PWID and having sex with them after using drugs.

Similar to the results from our KII and FGD, despite having knowledge about how to obtain injection materials, PWID in South Africa also reported difficulty in access [15]. A systematic review also found that stigma, distance and fear of criminalization due to drug use were reported to impact the access to safe injection materials, thus contributing to needle sharing behaviors [16]. A study conducted among PWID in South Africa also reported that the immediacy of drug need contributed to needle sharing because of the perceived lack of time required to go the pharmacy or hospital [15].

Participants mentioned structural barriers to access to health and social services such as distance, reluctance to appear in public services and fear of criminalization, as well as the identification requirement for the use of these services. These barriers were also reflected in research conducted in Tanzania where programs fail to reach those who could benefit because of requirements that make it difficult to enter and remain in the services [4].

While some participants reported the existence of key population friendly services and comprehensive services as facilitators to access and use of services, there was a general low awareness about the services currently available for PWID. Low awareness of services was also reported in the literature [16] and not surprisingly, one study found that health seeking behavior among PWID in Tanzania was directly correlated to knowledge of services [17], thus highlighting the importance of awareness campaigns targeting PWID about the existence of services.

Low quality of health services, including interaction of health care professionals and non-coordinated services [4] was also mentioned as a barrier to access among participants. In South Africa [14], a study reported that there is usually a long wait for access to both treatment as well as HIV testing, and generally, PWID feel that negative attitudes and unprofessional behavior from healthcare providers exacerbate the problem where they feel that they are treated with disrespect. Respondents mentioned the need to improve professionalism among staff of both HIV/ AIDS services as well as for staff in support and rehabilitation services, hiring more qualified personnel such as counselors and psychologists and that the existing personnel introduce themselves with a positive attitude and be careful and understanding towards clients [18].

Similar to our findings, previous studies have outlined various structural and environmental barriers to access to services including uncoordinated services, complicated requirements for treatment access, lack of transportation, high costs, and fear of stigmatization and criminalization [2, 4, 12, 13, 15, 16]; other important structural issues from the literature that were not mentioned in our study included homelessness and precarious housing [19]. These barriers to access and use of services can be addressed through the integration of HIV/PWID treatment services, peer education programs, enhanced training of health personnel to provide key population friendly services, community engagement, and the revision of criminal code which frames injection drug use as a public health issue [1, 4, 12, 16]. Many of these strategies can be a cost-effective particularly in resource-limited settings such as Mozambique [1].

### Limitations

Our findings represent the first exploration about access to and use of health and social services by PWID in Mozambique, however there are important limitations to consider. First, the KII and FGD were limited to specific urban geographic areas given the nature of the BBS surveys and therefore the results may not be generalized to other urban areas in Mozambique, nor to the realities of PWID residing in rural areas. Second, there are potential experiences and risk behaviors of some PWID subgroups, including women and people with higher income, that may not have been well represented in this assessment since the number of female participants and people of high income was virtually nonexistent.

Next, there was also potential selection bias because current or former PWID already engaged in services were more likely to have participated than PWID who are isolated from health and human services or support networks. Self-exclusion could have meant that more vulnerable subgroups may have declined to participate in FGD. However, we attempted to address this by ensuring that KII and FGD were continued until responses reached a level of saturation. Finally, given that injection drug use is a highly stigmatized and criminalized behavior in Mozambique, responses are potentially subject to information bias and social desirability bias. However, despite these limitations, the study provides important information about risk behaviors for HIV infection for PWID in Mozambique as well as their access and use of services.

## Conclusions

Understanding service delivery gaps identified during the Formative Assessment was used to strengthen referral systems during survey implementation. However, in general, the results helped to improve service delivery access and quality of services, both of which are necessary efforts aimed toward epidemic control in Mozambique. Interventions to support this key population must systematically address the barriers related to access to and use of health and social services [2]. Several policies and harm reduction programs must urgently be introduced such as detox and rehabilitation services, methadone treatment services, and prevention programs incorporating peer educators. Government-sponsored syringe exchange programs have the potential to reduce injection drug use behaviors in Mozambique; to date, there is only one needle and syringe exchange demonstration project. Finally, a patient-centered model of care, through the creation of mental health and psychiatric treatment services in public health facilities, rather than in-patient psychiatric facilities, is of the utmost importance. This model could ensure high-quality of services and also address the stigma and discrimination experienced by this population. In order for harm reduction efforts for PWID to be successful, a coordinated effort is necessary between health professionals, civil society, policy makers and donors.

### Recommendations

Further research is necessary to explore the specific gendered-experience of access and use of services by female PWID in Mozambique-education programs (Asher 2013).

## Data Availability

The dataset generated and/or analyzed during the current study are fully available at the Mozambique National Institute of Health (INS) data repository for researchers who meet the criteria for access to confidential data. Data are from the IBBS study’s whose authors may be contacted through: www.ins.gov.mz.

## Abbreviations

AIDS: Acquired Immunodeficiency Virus
BBS: Biological Behavioral Survey
CDC: Centre for Disease Control
FGD: Focus Group Discussion
HIV: Human Immunodeficiency Virus
IBBS: Integrated Biological Behavioral Survey
KII: Key Informant Interview
NGO: Non-Governmental Organization
PEFFAR: (United States) Presidents Emergency Plan For AIDS Relief
PWID: People Who Inject Drugs
UNODC: United Nations Office for Drug Control
US: United States
USD: United States Dollars
TB: Tuberculosis
